# Correction to: Intratumoral PD-1^high^ CD8^+^T cells correlate with AFP levels in HCC patients: a brief report

**DOI:** 10.1007/s13402-026-01176-8

**Published:** 2026-02-12

**Authors:** Zuzana Macek Jilkova, Julien Ghelfi, Lucile Dumolard, Christian Sengel, Bleuenn Brusset, Yann Teyssier, Charlotte Costentin, Thomas Decaens

**Affiliations:** 1 Univ. Grenoble Alpes, Institute for Advanced Biosciences, Research Center UGA/ Inserm U 1209 / CNRS 5309, Site Santé, Allée des Alpes, La Tronche, Grenoble, 38700 France; 2 Hepato-Gastroenterology and Digestive Oncology Department, CHU Grenoble Alpes, CS 10217, Grenoble, 38043 France; 3 Service de radiologie, CHU Grenoble Alpes, Grenoble, France

**Correction to: Cellular Oncology (2026) 49:39**

10.1007/s13402-026-01170-0

In this article the authors’ given and family names were interchanged. The author names Zuzana Macek Jilkova, Julien Ghelfi, Lucile Dumolard, Christian Sengel, Bleuenn Brusset, Yann Teyssier, Charlotte Costentin, Thomas Decaens were incorrectly written as Macek Jilkova Zuzana, Ghelfi Julien, Dumolard Lucile, Sengel Christian, Brusset Bleuenn, Teyssier Yann, Costentin Charlotte, Decaens Thomas.

The original article has been corrected.

